# Exites in Cambrian arthropods and homology of arthropod limb branches

**DOI:** 10.1038/s41467-021-24918-8

**Published:** 2021-07-30

**Authors:** Yu Liu, Gregory D. Edgecombe, Michel Schmidt, Andrew D. Bond, Roland R. Melzer, Dayou Zhai, Huijuan Mai, Maoyin Zhang, Xianguang Hou

**Affiliations:** 1 Yunnan Key Laboratory for Palaeobiology, Institute of Palaeontology, Yunnan University, Kunming, China; 2 MEC International Laboratory for Palaeobiology and Palaeoenvironment, Yunnan University, Kunming, China; 3 Department of Earth Sciences, The Natural History Museum, London, UK; 4 Bavarian State Collection of Zoology, Bavarian Natural History Collections, München, Germany; 5 Department Biology II, Ludwig-Maximilians-Universität München, Planegg-Martinsried, München Germany; 6 Department of Earth Sciences, Royal Holloway University of London, Egham, Surrey UK; 7 GeoBio-Center, Ludwig-Maximilians-Universität München, München, Germany

**Keywords:** Evolution, Evolutionary developmental biology, Palaeontology

## Abstract

The last common ancestor of all living arthropods had biramous postantennal appendages, with an endopodite and exopodite branching off the limb base. Morphological evidence for homology of these rami between crustaceans and chelicerates has, however, been challenged by data from clonal composition and from knockout of leg patterning genes. Cambrian arthropod fossils have been cited as providing support for competing hypotheses about biramy but have shed little light on additional lateral outgrowths, known as exites. Here we draw on microtomographic imaging of the Cambrian great-appendage arthropod *Leanchoilia* to reveal a previously undetected exite at the base of most appendages, composed of overlapping lamellae. A morphologically similar, and we infer homologous, exite is documented in the same position in members of the trilobite-allied Artiopoda. This early Cambrian exite morphology supplements an emerging picture from gene expression that exites may have a deeper origin in arthropod phylogeny than has been appreciated.

## Abstract (web summary)

The common ancestor of all living arthropods had biramous postantennal appendages, with an endopodite and exopodite branching off the limb base. This study uses microtomographic imaging of the Cambrian arthropod *Leanchoilia* to reveal a previously undetected exite at the base of most appendages, suggesting a deeper origin for exites in arthropod phylogeny.

## Introduction

Living arthropods are traditionally thought to have the appendages of their most recent common ancestor consisting of an antenna followed by a set of biramous post-antennal limbs^1^. Biramy refers to an inner branch (the endopodite) and outer branch (the exopodite), both of which arise from the same section of the limb base, known as the protopodite or basipodite. Correspondences between biramous appendages of crustaceans and Palaeozoic arthropods such as trilobites underpin the presumed homology of the endopodite, exopodite and protopodite and their origin at or deeper than the root of the arthropod crown group^1,2^. In this framework, some more proximal elements of the limb base, such as the coxa of crustaceans, are thought to have differentiated *de novo*^3^ or resulted from subdivision of an originally single part protopodite^4^.

The canonical view of homology between the two rami of biramous limbs across the arthropods was called into question when clonal analysis of peracarid pancrustaceans revealed that the endopodite and exopodite of their biramous limbs both form from growth zones along the main limb axis, and uniramous limbs result from a failure of this axis to split rather than from loss of the exopodite as traditionally thought^5,6^. This single axis contrasts with additional outgrowths from the limb base (known as exites), which grow along novel axes. This suggested that the biramous limb in crustaceans differs from the condition seen in many Cambrian arthropods, which was reinterpreted as a uniramous limb and an exite rather than an endopodite and exopodite, respectively^5^.

Fitting extant chelicerates into this framework is challenged by prosomal appendages generally being uniramous (composed of a telopodite widely homologised with an endopodite) and the opisthosomal appendages being modified into respiratory structures. Even when there is agreement on a theory of ancestral biramy in Chelicerata, the identification of particular structures as either exopodites or exites in chelicerates has been contentious. For example, the flabellum, a projection on leg VI of horseshoe crabs (Xiphosura), has been homologised with either an exopodite^1,2^ or an exite/epipodite^7,8^, and the same is true for the book gills^2,9^. Gene expression data even allow that the book gill opercula include a contribution from the walking leg^10^. Fossils assigned to the chelicerate stem^11^ (or crown^12^) group, such as *Offacolus*^13^ and *Dibasterium*^14^, have contributed to this debate because they have segmented rami that have been homologised with the endopodite and exopodite of biramous limbs^4^ and are inferred to have originated by splitting of a single axis^7^. A distinct identity for the supposed exopodite can nevertheless also be considered, as it appears to emerge from the body wall with distinct separation from the protopodite.

A different approach to evaluating homology of rami and exites is offered by functional studies of leg patterning genes. Comparison of leg gene function in the amphipod *Parhyale* and *Drosophila* and other insects serves to homologise the distal six podomeres of crustacean and insect appendages in a one-to-one manner. Combined with expression patterns of wing genes, morphologically varied exites can be associated with more proximal podomeres of the leg, even when they have been incorporated into the body wall^15^. This approach has been extended to chelicerates by drawing on the spider *Acanthoscurria*, concluding that chelicerate exites and the inferred exopodite of Silurian chelicerates such as *Dibasterium* and *Offacolus* are non-homologous with exopodites of pancrustaceans because they branch from different podomeres^7^.

In this work, we present evidence for a morphologically distinctive exite in Cambrian arthropods exposed by computed microtomography of fossils from the Cambrian (Series 2, Stage 3) Chengjiang Biota of Yunnan, China. Similarities between this exite in four species support its homology. Comparisons with other fossil and extant arthropods, in light of current phylogenetic frameworks, suggest an early origin of exites in arthropod evolution.

## Results and discussion

### A previously undetected exite in Cambrian arthropods

The structure of interest is best known from the megacheiran great-appendage arthropod *Leanchoilia illecebrosa*, in which it has been imaged on both sides of the body in the head and trunk. The exite of this species is illustrated and described in detail, with comparative accounts in another species of *Leanchoilia*, *L. obesa*, and in two members of the Artiopoda, *Naraoia spinosa* and *Retifacies abnormalis*. Artiopoda is a monophyletic group^16^ that unites trilobites with Palaeozoic taxa sharing a set of mostly homonomous post-antennal appendages of similar structure.

YKLP 11424 is a specimen of *Leanchoilia illecebrosa* of length 21 mm, preserved in lateral aspect (Supplementary Fig. 1a), complete apart from lacking the posterior portion of the telson. The great appendages, four additional cephalic appendage pairs, and nine preserved pairs of trunk appendages have been digitally dissected on both sides of the body (Supplementary Fig. 2). Most details of the protopodite, endopodite and exopodite of the biramous appendages correspond to previous descriptions of this species^17^ and the allied *L. superlata* from the Burgess Shale^18,19^ and a complete description is not presented.

**Fig. 1:**
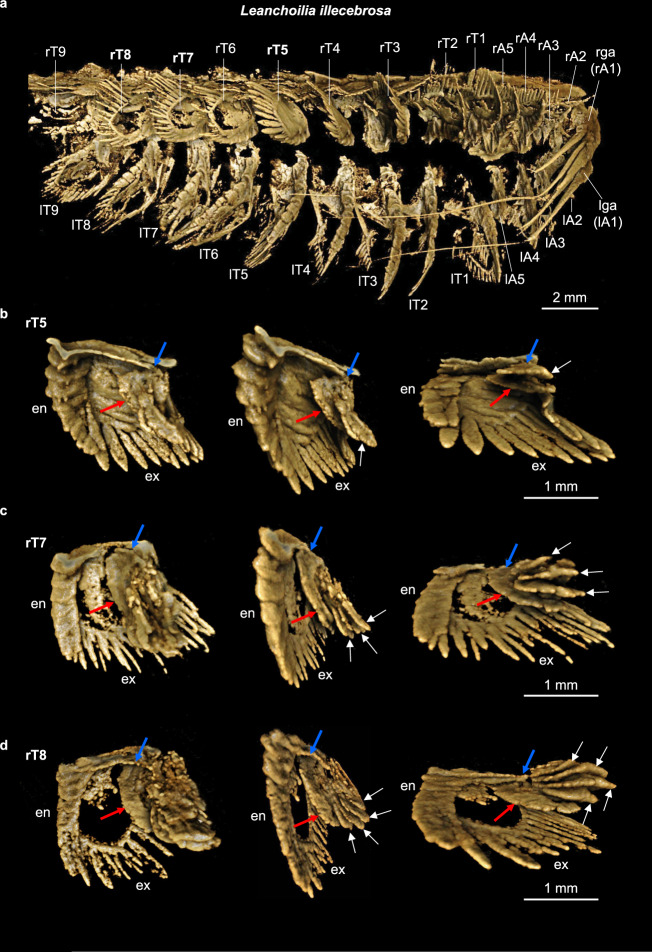
Computed tomographic images of YKLP 11424 showing selected exite-bearing appendages of *Leanchoilia illecebrosa*. **a** Ventral side of the animal. **b**–**d** Digitally dissected trunk appendages 5, 7, and 8 from the right side of the animal (rT5, rT7, rT8). Each appendage is shown at three different angles to demonstrate the endopodite (en), the exopodite (ex) and the exite consisting of one basal flap (red arrow) and several additional ones (white arrows). Blue arrows point to the attachment of the exite. Individual scale bars provided. A*n*, head appendage *n*; l, left; r, right; ga, great appendage; T*n*, trunk appendage *n*. Dissections of all appendages are available in Supplementary Figs. 2–4.

An outgrowth composed of two to five overlapping lamellae is observed at the proximal edge of the protopodite of the last two head appendages (Supplementary Fig. 3c,d) and in all trunk appendages (Fig. 1, Supplementary Fig. 4) of YKLP 11424. It is hereafter referred to as an exite based on its attachment to the protopodite proximal to that of the exopodite. The number of overlapping lamellae increases on more posterior appendages, there being two on the appendage of trunk segment 5, four on segment 7, and five on segment 8 (Fig. 1, Supplementary Fig. 4). Each lamella has a similar flap-like shape, and on each appendage a basal lamella attaches to the protopodite whereas the remaining, overlying lamellae each attach near the base of the basal lamella (Supplementary Movie 1). The specimen bears a morphologically similar exite in the same position on the fourth and fifth cephalic appendages and the first four pairs of trunk appendages, but its preservation is poorer than on the more posterior trunk segments. The more anterior appendages expose only fragments of lamellae (Supplementary Figs. 3c, d, 4a–d, Supplementary Movies 2, 3).

YKLP 11093 is a specimen of *Leanchoilia illecebrosa* of length 30 mm and is likewise preserved in lateral aspect (Supplementary Figs. 1b, 5), including the head and entire trunk. The four biramous cephalic appendages and first pair of trunk appendages are well preserved and have been digitally dissected from one side of the body. A lamellar exite is observed on the fourth (Supplementary Fig. 5d) and fifth (Supplementary Fig. 5e) cephalic appendages and on the first trunk appendage (Supplementary Fig. 5f, Supplementary Movie 4), in each case being composed of flap-like lamellae. The lamellae are best preserved on the first trunk appendage, in which they consist of the basal lamella and two overlapping lamellae. The margins of the exite lamellae are fringed by a few setae (Supplementary Fig. 5d), which are well shown by additional specimens (YKLP 10938, 11089; see below). A series of appendages shows the exopodite folded along the hinge by which it attaches to the protopodite, such that the exite comes to lie between the endopodite and exopodite (Supplementary Fig. 6). The endopodite, exopodite and exite are each oriented in a different plane, rejecting the possibility that the exite attaches to the exopodite rather than having an independent attachment to the protopodite.

Information from the two specimens thus indicates a consistent appearance of the lamellae on the last two cephalic appendages but suggests minor variation in the number of lamellae on anterior trunk appendages (i.e., two versus three), potentially related to differences in body size.

YKLP 10938 and 11089 are two complete specimens of *L. illecebrosa* in lateral aspect. The exites are visible on posterior cephalic and anterior trunk appendages in light microscopy (Supplementary Fig. 7), revealing up to nine setae of variable length fringing the margin of a lamella (Supplementary Fig. 7c).

**Fig. 2:**
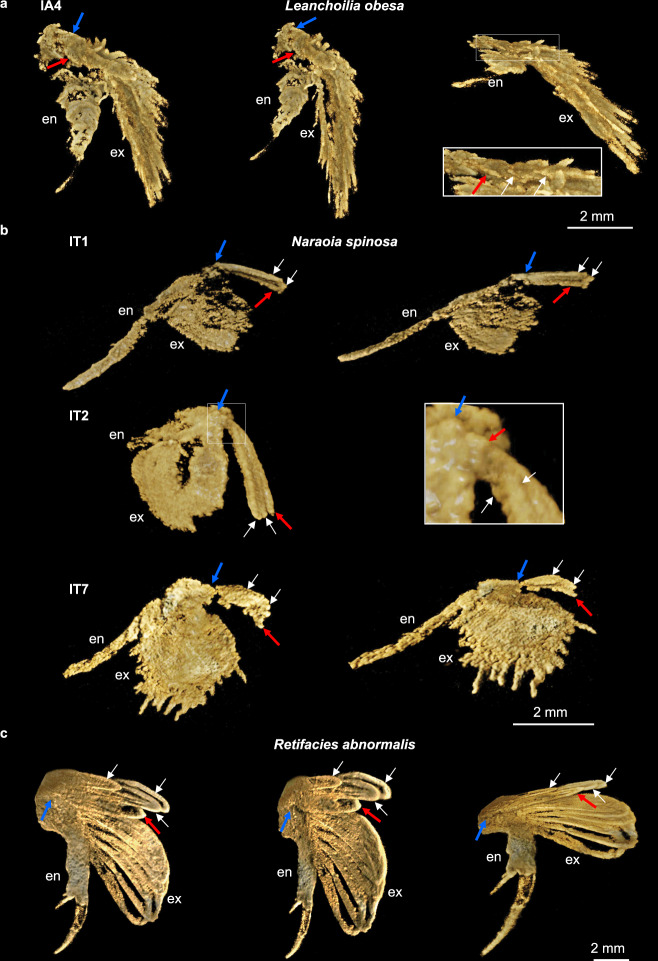
Computed tomographic images showing exite-bearing appendages. **a** *Leanchoilia obesa* (YKLP 13323). **b** *Naraoia spinosa* (YKLP 11425). **c** *Retifacies abnormalis* (YKLP 11426). Each appendage is shown at different angles to demonstrate the endopodite (en), the exopodite (ex) and the exite consisting of one basal flap (red arrow) and several additional ones (white arrows). Blue arrows point to the attachment of the exite. Individual scale bars provided. A*n*, head appendage *n*; l, left; r, right; T*n*, trunk appendage *n*. CT images of the entire specimens are available in Supplementary Fig. 8.

YKLP 13323 is a complete specimen of *Leanchoilia obesa*, the holotype of the species, preserved in dorsal view (Supplementary Fig. 1c). An appendage from the head (lA4 in Supplementary Fig. 8a) has been digitally dissected (Fig. 2a, Supplementary Movie 5) to reveal very similar morphology to exites on cephalic and trunk appendages of *L. illecebrosa*. An exite attaches at the same position on the protopodite, composed of a basal lamella and two additional flap-like lamellae overlapping it (Fig. 2a inset). Each of these overlying lamellae attaches proximally, but their exact attachment is less clear in this species.

YKLP 11425 is a complete specimen of *Naraoia spinosa* with the head shield folded at a high angle relative to the trunk shield, as is common in this species (Supplementary Figs. 1d, 8b). Three trunk appendages, lT1, lT2 and lT7 (Supplementary Fig. 8b), have been digitally dissected (Fig. 2b, Supplementary Movie 6) and reveal exites similar to those of *Leanchoilia*. An outgrowth consisting of three overlapping lamellae affixes to the proximal-most portion of the protopodite, narrowly overlapping the exopodite. One of these exites has relatively short and wide lamellae, whereas the better-preserved ones have longer, narrower lamellae that all terminate at almost the same length. The exite lamellae become shorter and broader posteriorly. The basal lamella attaches to the protopodite whereas the overlying lamellae each attach near the base of the basal lamella (Fig. 2b).

YKLP 11426 is a fragmentary specimen of *Retifacies abnormalis*, preserving only the trunk (Supplementary Figs. 1e, 8c, 9a,b), but is confidently identified by diagnostic reticulate sculpture on the tergum, pygidial shape, and a styliform, annulated telson. Several trunk appendages bear proximal outgrowths from the protopodite (Supplementary Fig. 8c). One of the appendages has been digitally dissected (Fig. 2c, Supplementary Movie 7) to reveal a similar situation seen in *Leanchoilia* and *Naraoia*. Four lamellae of various sizes originate from the proximal portion of the protopodite. The lamellae are elongate and paddle-shaped, of similar morphology to both each other and to the exopodite lamellae. The lamellae are imbricated with the basal lamella offset distally, and the overlying lamellae being grouped more proximally. Each lamella attaches to the protopodite independently.

**Fig. 3:**
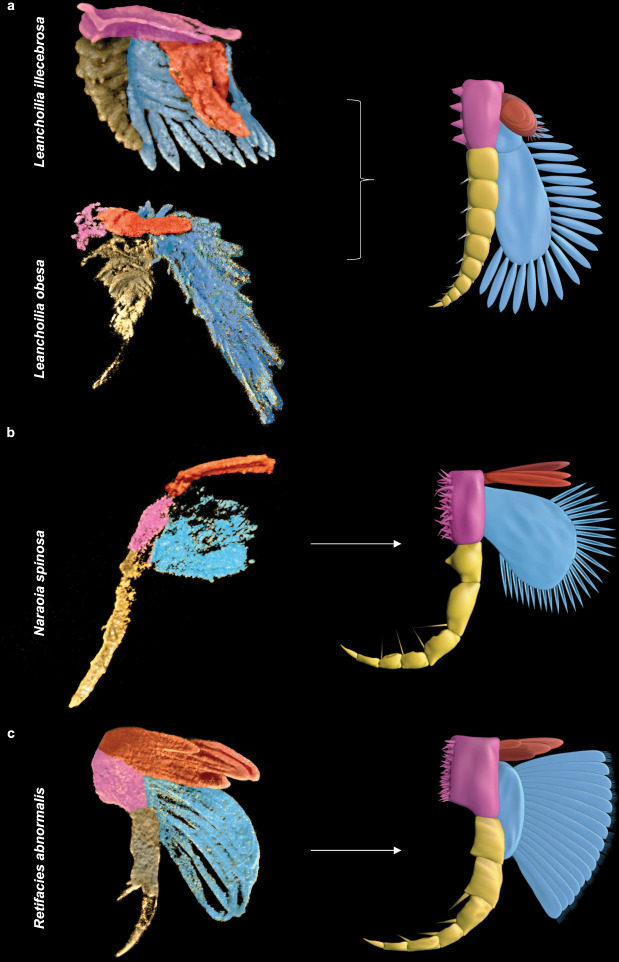
Three-dimensional models of exite-bearing appendages. **a** *Leanchoilia illecebrosa* and *Leanchoilia obesa*. **b** *Naraoia spinosa*. **c** *Retifacies abnormalis*. Protopodite, endopodite and exopodite are coloured in pink, yellow and blue, respectively. Exite is in red and has not previously been detected. Not to scale. Digitally dissected protopodite, endopodite, exopodite, and exite are shown in Supplementary Fig. 10.

The exites of *Leanchoilia*, *Naraoia* and *Retifacies* resemble each other in being composed of a few (two to five) overlapping lamellae and emerging from the proximal portion of the protopodite (Fig. 3, Supplementary Fig. 10). An additional shared character of *Leanchoilia* and *Naraoia* is that the basal lamella forms the attachment to the protopodite and the overlapping lamellae branch from the basal lamella (Fig. 3). The lamellae of the exite of *Retifacies* resemble those of the exopodite in both having a similar paddle-like shape, but in all three cases the exite is regarded as a discrete structure rather than being an outgrowth of the exopodite, like the divided exopodite called a pseudepipodite in cephalocarids^20^. *Naraoia* in particular shows a clear separation of the attachment of the exite and exopodite and a marked difference in the morphology of the two branches.

### Exites have multiple origins

Among the varied kinds of exites known in arthropods, comparisons with Cambrian fossils have mostly focused on the epipodites of crustaceans, which can unequivocally be distinguished from the exopodite of a limb when both are present (as is likewise the case in the Cambrian species studied here). Epipodites are unmusculated flaps or clubs originating proximal to the exopodite on the coxal or precoxal part of the protopodite of post-maxillulary appendages^4,21^, serving an osmoregulatory or respiratory function^22^. Based on differences in position and morphology, exites and even more specifically epipodites have been ascribed multiple independent origins within Pancrustacea^1^, although some correspondences in gene expression are consistent with their homology between such divergent groups as branchiopods and malacostracans^4^. The pancrustacean *Yicaris dianensis* from Cambrian Series 2, Stage 3^23^ figures prominently in discussion about the timing of origin of epipodites. A series of leaf-shaped exites in *Yicaris* has been interpreted as epipodites^22,23^ or as exites of an independently evolved nature^4,21^. Irrespective of this debate, leaf-shaped epipodites on the biramous trunk appendages of *Ercaicunia multinodosa*, a stem-group pancrustacean, attest to their origin by Cambrian Stage 3^24^.

The absence of epipodites in some lineages of Pancrustacea weakens the case for epipodites being so deeply nested in Mandibulata that the exites in *Leanchoilia* and Artiopoda are their homologues, although *Ercaicunia* suggests an earlier origin in Pancrustacea than is predicted by extant lineages on their own^24^. The likely multi-fold derivation of epipodites within Pancrustacea and the discovery of a previously undetected exite with a distinctive lamellar structure in distantly allied Cambrian arthropods are most compatible with epipodites and lamellar exites having independent evolutionary origins. However, it is emerging that exites are more common in arthropods than is commonly assumed. In addition to the flabellum of xiphosuran chelicerates noted above, expression of leg patterning genes and wing genes suggests that the coxal and tergal plates of amphipod crustaceans, paratergal outgrowths and tracheae of insects, and wings of pterygote insects are modified exites^7,15^. Adding the fossil data presented here, which cover previously unsampled arthropod lineages, exites may have a deeper origin in arthropod phylogeny than has been thought.

### Implications for exopodites and exites in chelicerates

Interpreting the phylogenetic significance of similarities between Megacheira and Artiopoda is complicated by both groups repeatedly being placed in two different positions on the arthropod tree. Megacheira is resolved by character argumentation or quantitative phylogenetic analyses as either stem-group Euarthropoda^25,26^ or as stem-group Chelicerata^27–30^. Trilobites and other artiopodans are alternatively allied to Mandibulata^31–34^ or to Chelicerata^26,35^. The latter is consistent with the historical Arachnomorpha^36,37^ or Lamellipedia^38^ hypotheses, the latter named for lamellate setal blades in the exopodite. The evidence for homology of the lamellar exites in Megacheira (*Leanchoilia*) and Artiopoda (*Naraoia*, *Retifacies*) presented here suggests a single origin of this trait and accordingly, assuming that this trait is apomorphic, a close phylogenetic affinity between these taxa. Since both groups have been allied with chelicerates and both are recovered in some phylogenetic analyses in the chelicerate stem-group^35^, this character may serve as a synapomorphy of a clade within total-group Chelicerata. An alternative interpretation would be that this kind of exite is a symplesiomorphy, potentially retained from flaps bearing setal blades in stem-group arthropods such as *Opabinia*^39^. However, *Leanchoilia* and *Naraoia* shared detailed similarity in the mode of growth of overlapping lamellae via the attachment of overlying lamellae to the basal lamella, so a symplesiomorphy interpretation would force an exite composed of a few overlapping lamellae to be present throughout the arthropod stem-group/crown-group transition but to have gone undetected.

The likely attachment of the exopodite of the chelicerates *Dibasterium* and *Offacolus* to the body wall rather than to the protopodite noted above may be approximated in trilobites. It has recently been argued that the exopodite of the Cambrian trilobite *Olenoides serratus* attaches partly to the body wall and partly to the limb base^40^. In conjunction with previous work, which suggests that exopodite and endopodite form by the splitting of the main leg axis^5,6^ and that exopodites of different arthropod groups originate on different podomeres^7^, these findings reinforce the argument that the trilobite exopodite may not be homologous with the pancrustacean exopodite.

Our proposal that a lamellar exite is homologous in Artiopoda and Megacheira carries a prediction that a corresponding exite is present in other representatives of these groups, as well as in lineages separating them in the phylogeny. Microtomography of early-derived trilobites and stem-group chelicerates offers potential for discovering additional instances of this structure. Palaeontological data for additional morphologies of exites add to the emerging picture from gene expression in extant arthropods that exites are a more pervasive source of evolutionary novelty in appendage form than has been appreciated.

## Methods

### Material

*Leanchoilia illecebrosa* (YKLP 11424, YKLP 11093), *Naraoia spinosa* (YKLP 11425): Collected from Yu’anshan Member, Chiungchussu Formation, *Eoredlichia*-*Wutingaspis* trilobite biozone, Cambrian Series 2, Stage 3, Mafang village, Haikou county, Kunming, Yunnan, China (24°46'20” N, 102°35'10” E). *Leanchoilia obesa* (YKLP 13323), *Retifacies abnormalis* (YKLP 11426, YKLP 11430): Collected from Yu’anshan Member, Chiungchussu Formation, *Eoredlichia*-*Wutingaspis* trilobite biozone, Cambrian Series 2, Stage 3, Ercaicun village, Haikou county, Kunming, Yunnan, China (24°47’ N, 102°34’ E).

### Microscopic observation and documentation

Fossils were observed and prepared under a Leica M205 microscope. Macrophotography shown in Supplementary Figs. 1, 7, and 9 was first undertaken using a digital camera (Olympus E-20P) linked to a microscope Leica MZ12, and, for higher resolution, repeated with a Canon EOS 5DSR camera (DS126611) coupled with a MP-E 65 mm macro photo lens, illuminated with a LEICA LED5000 MCITM. Similar results were obtained, and the higher resolution imagery was used in this work. Images were processed with Adobe Photoshop CC 2018 and arranged into figures with Microsoft Office 2016.

### Micro-computed tomography and 3D rendering

All specimens were scanned with a Micro-X ray-CT: Xradia 520 Versa (Carl Zeiss X-ray Microscopy, Inc., Pleasanton, USA). Except for YKLP 13323 (*Leanchoilia obesa*; scanned at the Institute of Geology and Geophysics, Chinese Academy of Sciences), all other specimens were scanned at the Yunnan Key Laboratory for Palaeobiology, Institute of Palaeontology, Yunnan University, Kunming, China.

Scanning parameters are as follow: *Leanchoilia illecebrosa* (YKLP 11424): Beam strength: 50 kV/4wW, no Filter, Resolution: 8.92 µm, Number of TIFF images: 2558. *Leanchoilia illecebrosa* (YKLP 11093): Beam strength: 50 kV/4 W, no Filter, Resolution: 9.90 µm, Number of TIFF images: 3390. *Leanchoilia obesa* (YKLP 13323): Beam strength: 70 kV/6 W, no Filter, Resolution: 9.99 µm, Number of TIFF images: 2030. *Naraoia spinosa* (YKLP 11425): (1) For the entire specimen: Beam strength: 60 kV/5 W, no Filter, Resolution: 11.87 µm, Number of TIFF images: 1974; (2) For the appendages: Beam strength: 70 kV/6 W, no Filter, Resolution: 6.22 µm, Number of TIFF images: 2362. *Retifacies abnormalis* (YKLP 11426): (1) For the entire specimen: Beam strength: 60 kV/5 W, Filter: LE4, Resolution: 27.45 µm, Number of TIFF images: 2534; (2) For the appendages: Beam strength: 60 kV/5 W, no Filter, Resolution: 17.01 µm, Number of TIFF images: 3864. *Retifacies abnormalis* (YKLP 11430): Beam strength: 70 kV/6 W, Filter: LE4, Resolution: 17.39 µm, Number of TIFF images: 1014. All TIFF images were imported into the software Drishti (Version 2.4) to generate 3D models and enable digital dissections of various structures. Images were captured with the same software, processed with Adobe Photoshop CC 2018, and arranged into figures with Microsoft Office 2016. 3D reconstructions shown in Fig. 3 were produced in Blender 2.90.

### Reporting summary

Further information on research design is available in the Nature Research Reporting Summary linked to this article.

## Supplementary information

Supplementary Information

Description of Additional Supplementary Files

Supplementary Movie 1

Supplementary Movie 2

Supplementary Movie 3

Supplementary Movie 4

Supplementary Movie 5

Supplementary Movie 6

Supplementary Movie 7

Reporting Summary

## Data Availability

The authors declare that all data supporting the findings of this study are available within the article. The raw CT data generated in this study have been deposited in Zenodo (open access) [10.5281/zenodo.4782778].
