# Supplementary material for: Exites in Cambrian arthropods and homology of arthropod limb branches
Source: Nat Commun. 2021 Jul 30;12:4619. doi: 10.1038/s41467-021-24918-8 (PMC8324779; doi:10.1038/s41467-021-24918-8)
Supplement: Supplementary file 1 — Supplementary Information [file 41467_2021_24918_MOESM1_ESM.pdf]

Exites in Cambrian arthropods and homology of arthropod limb branches

Supplementary information

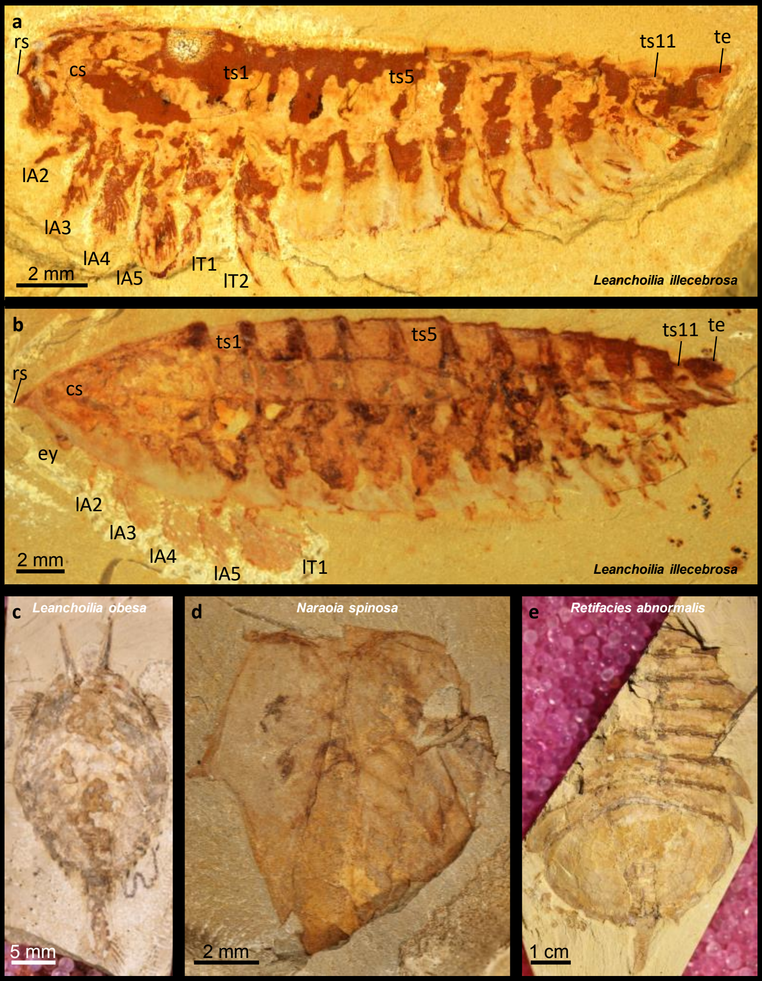

Supplementary Fig. 1 Light photographs of the specimens studied. a *Leanchoilia illecebrosa* (YKLP 11424). **b** *Leanchoilia illecebrosa* (YKLP 11093, horizontally flipped). **c** *Leanchoilia obesa* (YKLP 13323). **d** *Naraoia spinosa* (YKLP 11425). **e** *Retifacies abnormalis* (YKLP 11426). Individual scale bars provided. A*n*, cephalic appendage *n*; cs, cephalic shield; ga, great appendage; l, left; r, right; rs, rostrum; te, telson; ts*n*, trunk segment *n*; T*n*, trunk appendage *n*.

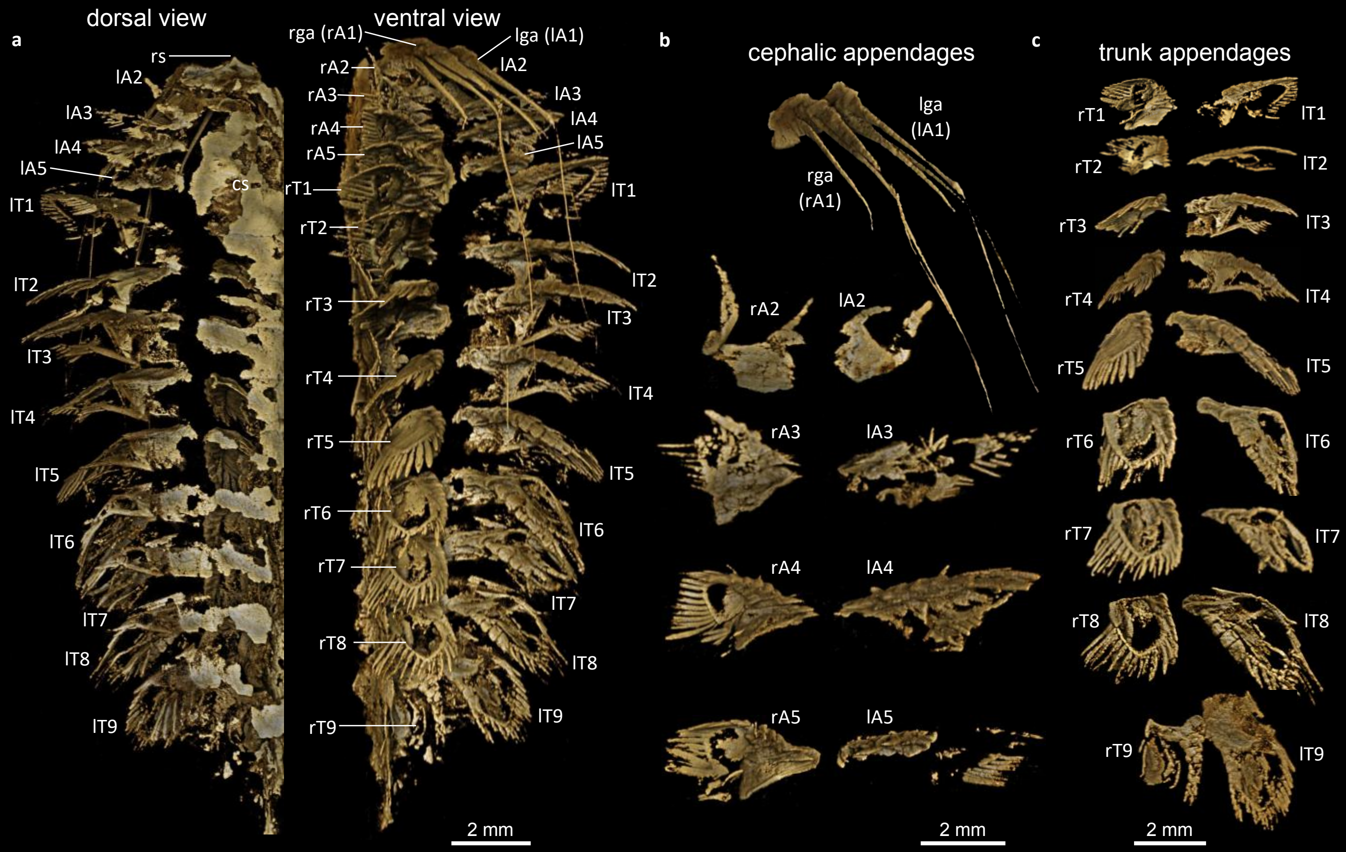

Supplementary Fig. 2 Computed tomographic images of YKLP 11424 showing the entire body and all dissected appendages of *Leanchoilia illecebrosa*. a CT images showing the dorsal and ventral sides of YKLP 11424. **b** Digitally dissected cephalic appendages. **c** Dissected trunk appendages. Individual scale bars provided. A*n*, head appendage *n*; l, left; r, right; rs, rostrum; ga, great appendage; T*n*, trunk appendage *n*.

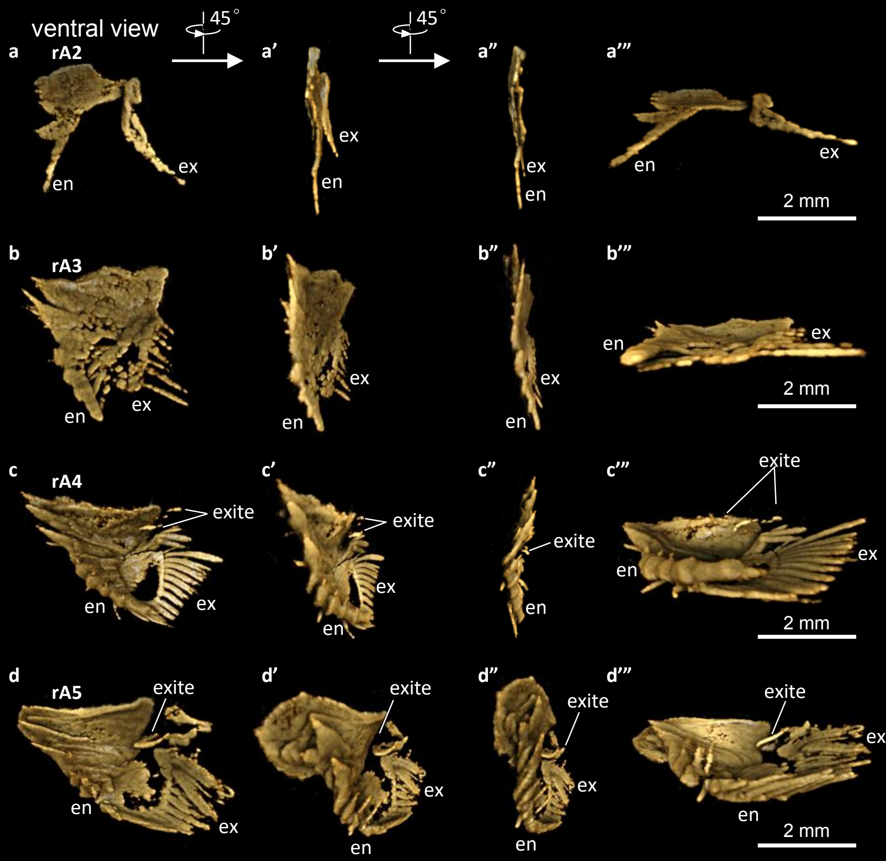

Supplementary Fig. 3 Computed tomographic images showing different angles of the dissected biramous appendages in the head of *Leanchoilia illecebrosa* (YKLP 11424). a-d Horizontally flipped images of the biramous appendages shown in Supplementary Fig. 2b. Viewed from the ventral side of the animal. **a'-d'** 45°rotation of a-d. **a"-d"** 45°rotation of a'-d'. **a"'-d"'** appendages viewed from their tips. Note that exite is not observed on cephalic appendages 2 and 3 (rA2, rA3) but is poorly preserved on the two subsequent ones (rA4, rA5). Individual scale bars provided. A*n*, cephalic appendage *n*; en, endopodite; ex, exopodite; r, right.

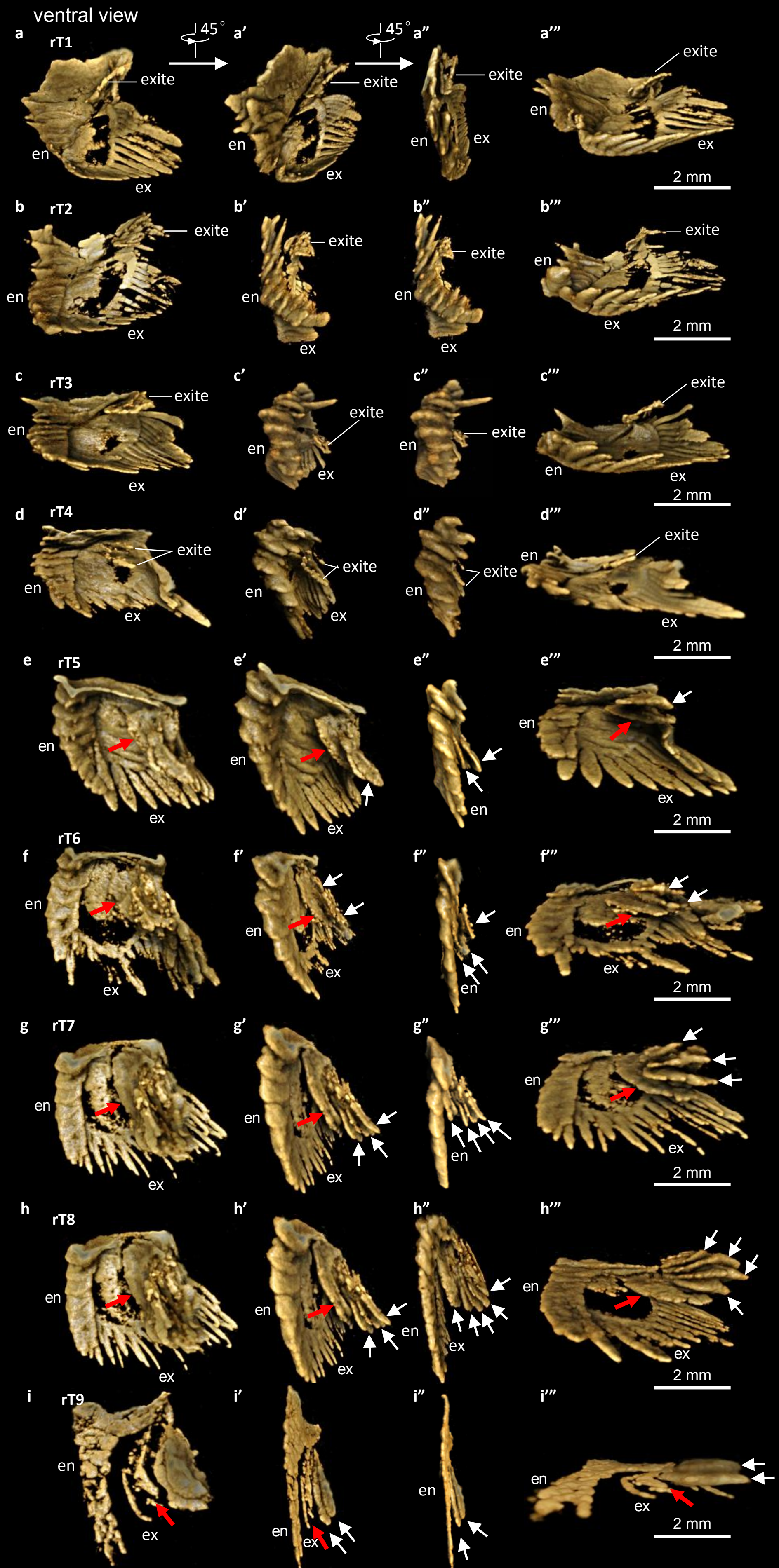

Supplementary Fig. 4 Computed tomographic images showing different angles of the dissected biramous appendages in the trunk of *Leanchoilia illecebrosa* (YKLP 11424). a-i Horizontally flipped images of the biramous appendages shown in Supplementary Fig. 2c. Viewed from the ventral side of the animal. **a'-i'** 45°rotation of a-i. **a"-i"** 45°rotation of a'-i'. **a'"-i'"** appendages viewed from their tips. Note the poorly-preserved exite in trunk appendages 1-4 (rT1–rT4). A well-preserved exite with one basal flap (red arrow) and several additional flaps (white arrows) are observed in trunk appendages 5-9 (rT5–rT9). Individual scale bars provided. A*n*, cephalic appendage *n*; en, endopodite; ex, exopodite; r, right.

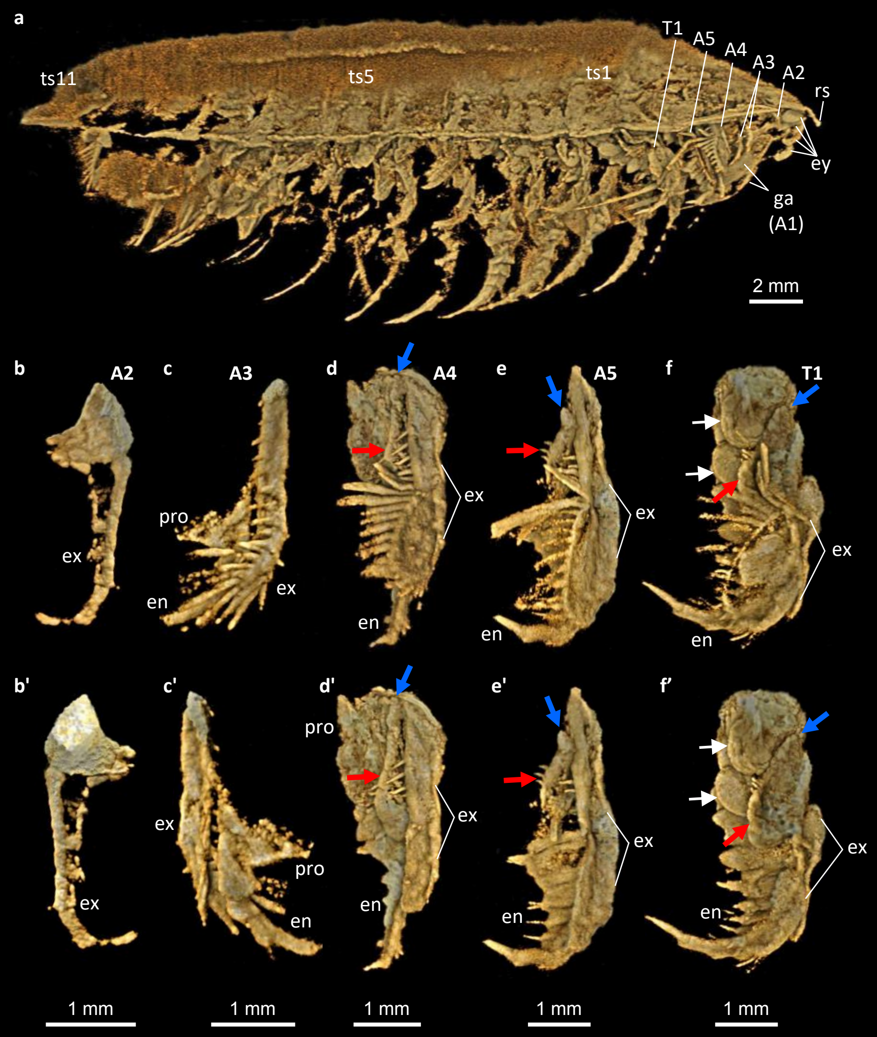

Supplementary Fig. 5 Computed tomographic images of YKLP 11093 showing selected appendages of *Leanchoilia illecebrosa*. **a** Lateral side of the animal. **b**–**f** Digitally dissected biramous appendages from the head (A2–A5) and the first trunk segment (T1). **b'**, **c'** 180-degree rotation of **b** and **c**, showing the other side of A2 and A3, respectively. **d'**–**f'** A4, A5 and T1 with their exopodite partly removed, exposing the complete exite. Note the absence of an exite in cephalic appendages 2 and 3 (A2, A3). A well-preserved exite with one basal flap (red arrow) is preserved in A4, A5 and T1, and several additional flaps (white arrows) are preserved in T1. Blue arrows point to the attachment of the exite. Individual scale bars provided. A*n*, cephalic appendage *n*; en, endopodite; ex, exopodite; ey, eye; ga, great appendage; pro, protopodite; rs, rostrum; T*n*, trunk appendage *n*; ts*n*, trunk segment *n*. All images are horizontally flipped.

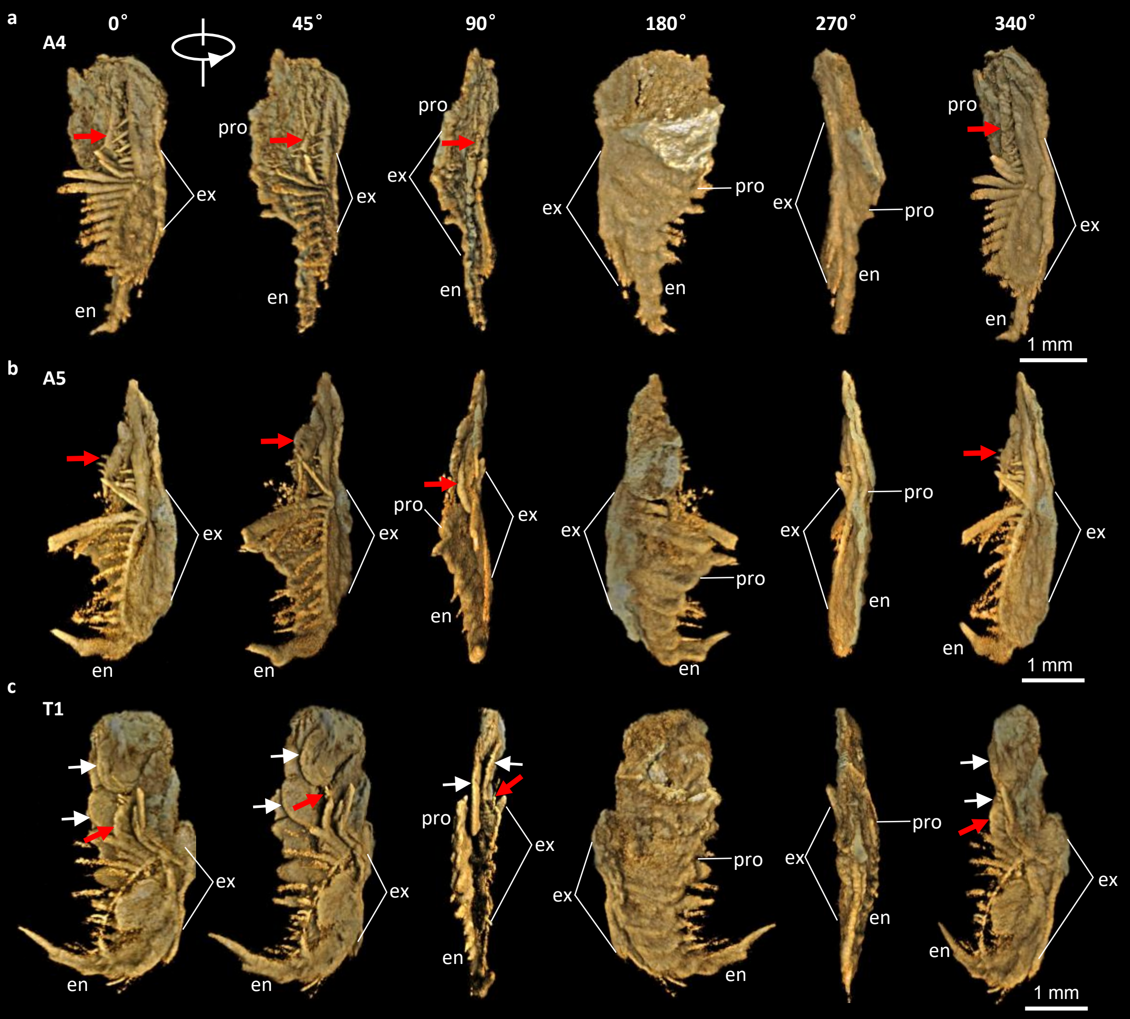

Supplementary Fig. 6 Computed tomographic images of YKLP 11093 showing selected exite-bearing appendages of *Leanchoilia illecebrosa*. a–c Comparable angles of A4, A5 and T1 showing the folding of exopodite along the hinge between the exopodite and the endopodite. Note that the exite is attached to the limb at a more proximal position than the exopodite (45 degrees and 90 degrees). Individual scale bars provided. A*n*, cephalic appendage *n*; en, endopodite; ex, exopodite; pro, protopodite. All images are horizontally flipped.

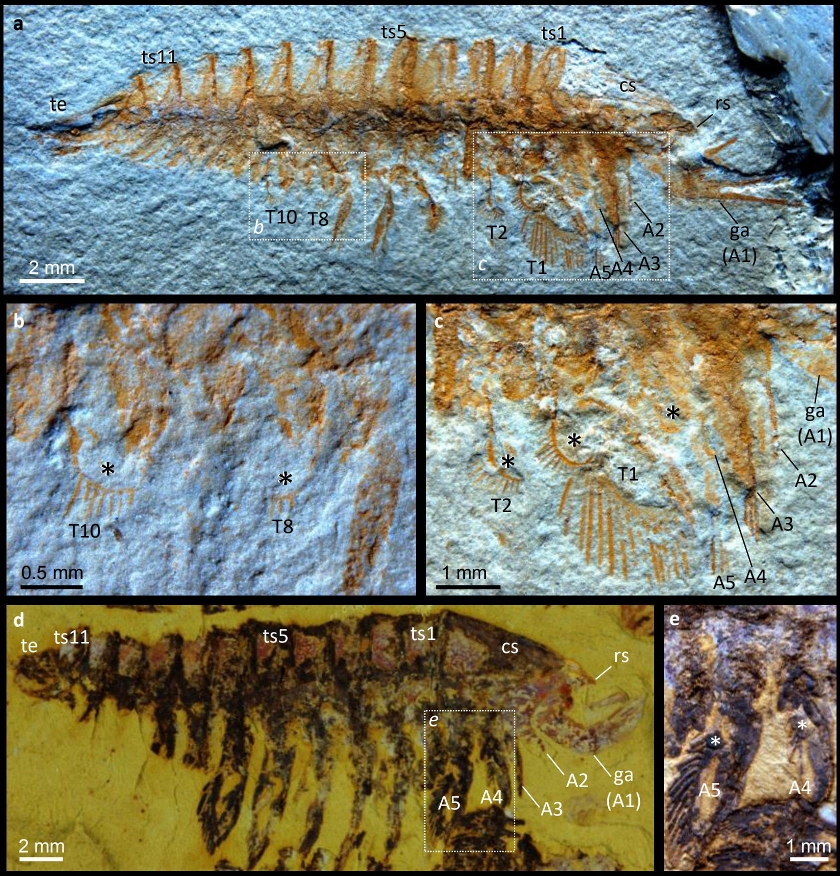

Supplementary Fig. 7 Light microscopic images showing proximal exites (asterisks) on the appendages of *Leanchoilia illecebrosa*. **a–c** YKLP10938, **d, e** YKLP11089. Individual scale bars provided. A*n*, head appendage *n*; cs, cephalic shield; ga, great appendage; rs, rostrum; te, telson; ts*n*, trunk segment *n*; T*n*, trunk appendage *n*.

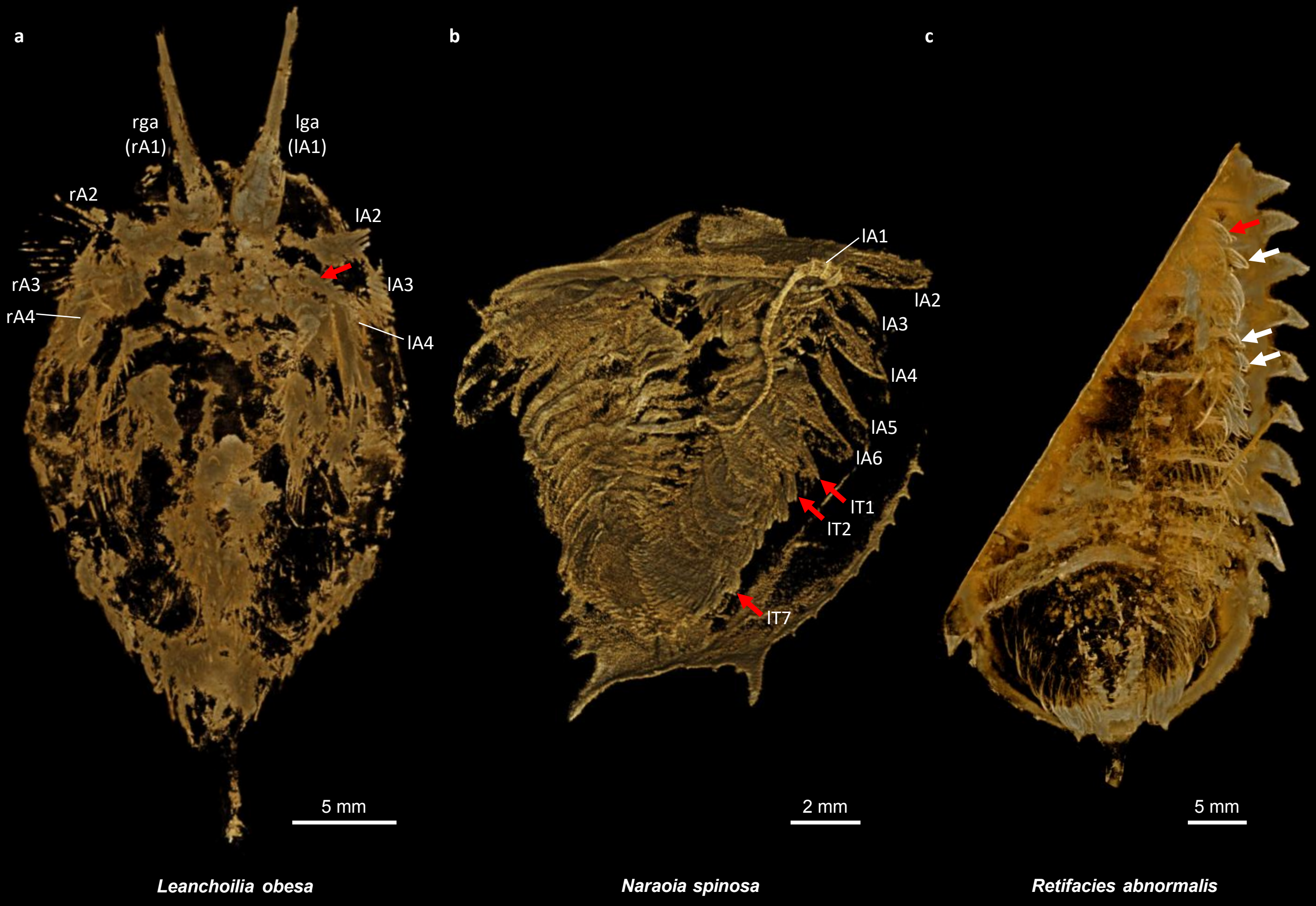

Supplementary Fig. 8 Computed tomographic images showing the ventral side of the studied specimens. a *Leanchoilia obesa* (YKLP 13323). **b** *Naraoia spinosa* (YKLP 11425). **c** *Retifacies abnormalis* (YKLP 11426). Red arrows point to the dissected appendages shown in Figure 2, while white arrows point to other exite-bearing appendages in the specimens. Individual scale bars provided. A*n*, cephalic appendage *n*; ga, great appendage; l, left; r, right; T*n*, trunk appendage *n*.

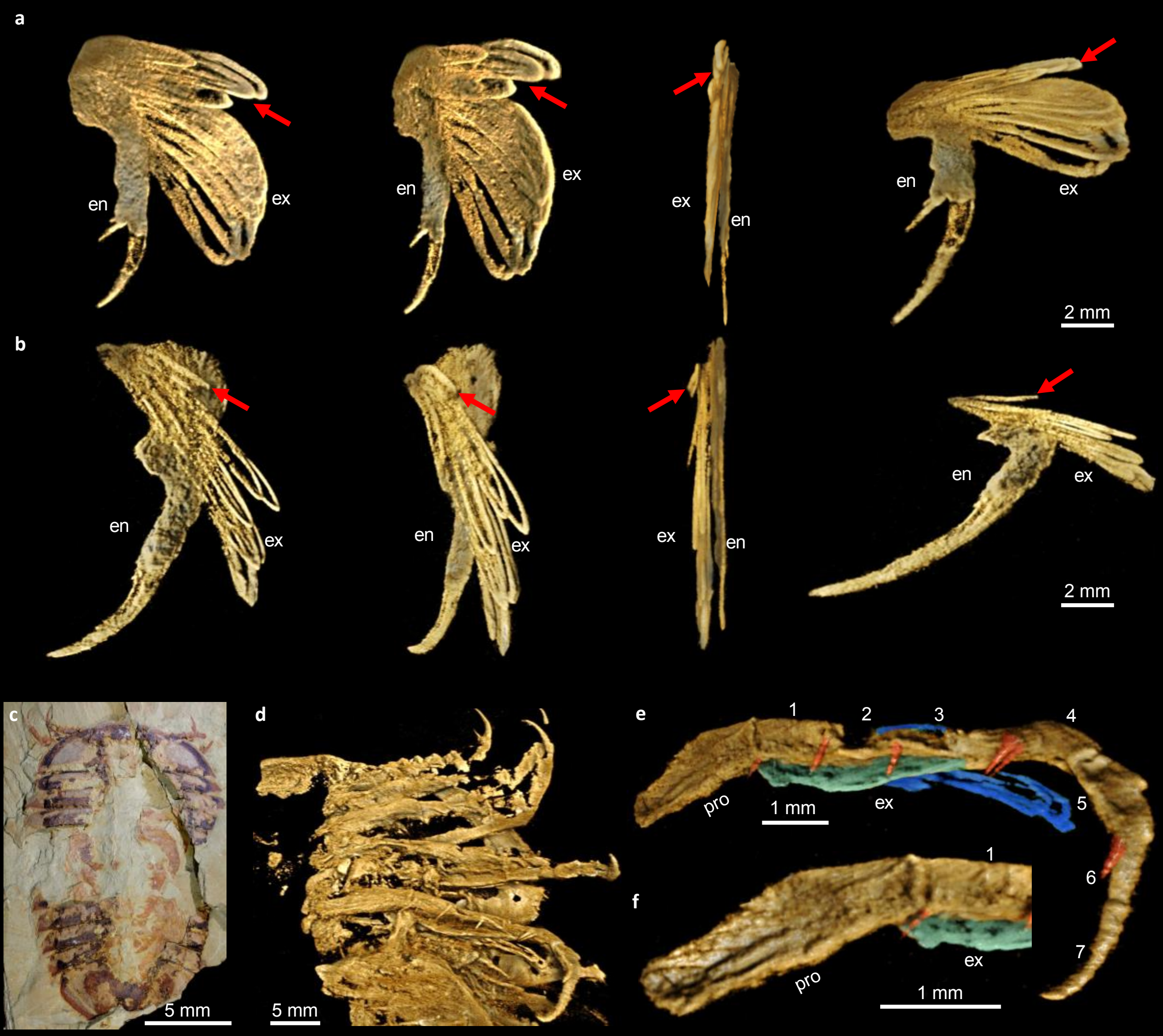

Supplementary Fig. 9 Computed tomographic images showing dissected appendages of *Retifacies abnormalis*. a, b Different angles of exite-bearing (red arrows) biramous appendages in the trunk of YKLP 11426. **c** Specimen YKLP 11430. **d** Ventral side of the head region of YKLP 11430. **e** Dissected appendage showing morphological details in the endopodite, exopodite and protopodite. Red colour highlights the medial spines of each endopodite podomere, cyan and blue label the exopodite flap and its marginal setae, respectively. **f** Close-up of the protopodite shown in **e**. Individual scale bars provided. en, endopodite; ex, exopodite; *n*, endopodite podomere *n*; pro, protopodite.

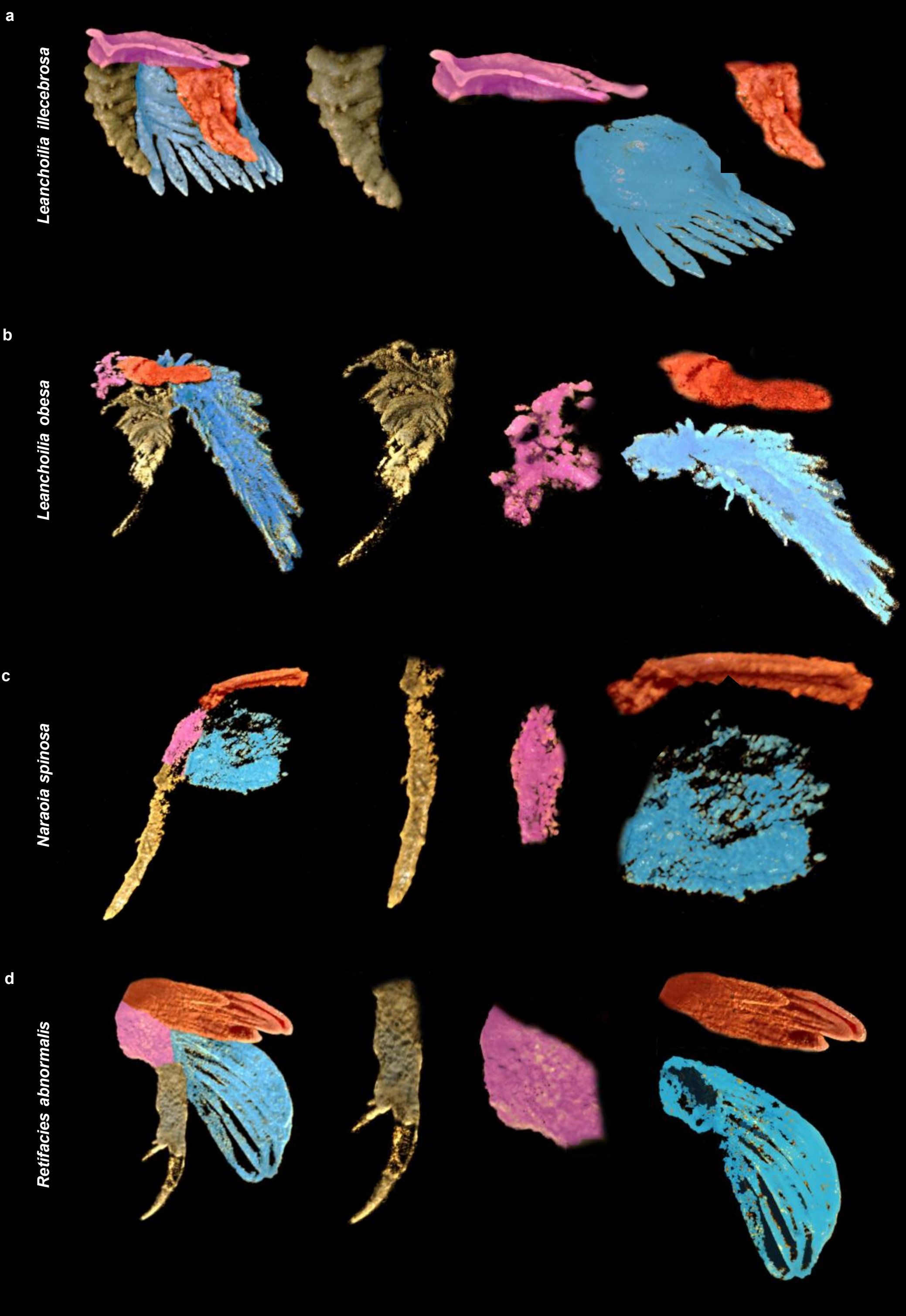

Supplementary Fig. 10 Computed tomographic images showing digitally dissected elements of the appendages. Endopodite (yellow), exopodite (blue), protopodite (pink) and exites (red) of *Leanchoilia illecebrosa* (**a**), *L. obesa* (**b**), *Naraoia spinosa* (**c**), and *Retifacies abnormalis* (**d**). Not to scale.
