# Supplementary material for: Exites in Cambrian arthropods and homology of arthropod limb branches
Source: Nat Commun. 2021 Jul 30;12:4619. doi: 10.1038/s41467-021-24918-8 (PMC8324779; doi:10.1038/s41467-021-24918-8)
Supplement: Supplementary file 2 — Description of Additional Supplementary Files [file 41467_2021_24918_MOESM2_ESM.docx]

**Exites in Cambrian arthropods and homology of arthropod limb branches**

**Supplementary files**

**Supplementary Movie 1 - main figure 1b**

**Supplementary Movie 2 – supplementary figure 3c**

**Supplementary Movie 3 – supplementary figure 3d**

**Supplementary Movie 4 – supplementary figure 5f**

**Supplementary Movie 5 - main figure 2a**

**Supplementary Movie 6 - main figure 2b**

**Supplementary Movie 7 - main figure 2c**
