# Supplementary material for: Exites in Cambrian arthropods and homology of arthropod limb branches
Source: Nat Commun. 2021 Jul 30;12:4619. doi: 10.1038/s41467-021-24918-8 (PMC8324779; doi:10.1038/s41467-021-24918-8)
Supplement: Supplementary file 10 — Reporting Summary [file 41467_2021_24918_MOESM10_ESM.pdf]

nature research

Corresponding author(s): Xianguang Hou and Gregory D. Edgecombe

Last updated by author(s): 28/06/21

Reporting Summary

Nature Research wishes to improve the reproducibility of the work that we publish. This form provides structure for consistency and transparency in reporting. For further information on Nature Research policies, see our Editorial Policies and the Editorial Policy Checklist.

Statistics

For all statistical analyses, confirm that the following items are present in the figure legend, table legend, main text, or Methods section.

n/a	Confirmed	
☒	☐	The exact sample size (n) for each experimental group/condition, given as a discrete number and unit of measurement
☒	☐	A statement on whether measurements were taken from distinct samples or whether the same sample was measured repeatedly
☒	☐	The statistical test(s) used AND whether they are one- or two-sided<br>*Only common tests should be described solely by name; describe more complex techniques in the Methods section.*
☒	☐	A description of all covariates tested
☒	☐	A description of any assumptions or corrections, such as tests of normality and adjustment for multiple comparisons
☒	☐	A full description of the statistical parameters including central tendency (e.g. means) or other basic estimates (e.g. regression coefficient) AND variation (e.g. standard deviation) or associated estimates of uncertainty (e.g. confidence intervals)
☒	☐	For null hypothesis testing, the test statistic (e.g. F, t, r) with confidence intervals, effect sizes, degrees of freedom and P value noted<br>*Give P values as exact values whenever*
☒	☐	For Bayesian analysis, information on the choice of priors and Markov chain Monte Carlo settings
☒	☐	For hierarchical and complex designs, identification of the appropriate level for tests and full reporting of outcomes
☒	☐	Estimates of effect sizes (e.g. Cohen's d, Pearson's r), indicating how they were calculated

Our web collection on statistics for biologists contains articles on many of the points above.

Software and code

Policy information about availability of computer code

Data collection	Drishti (Version 2.4)
Data analysis	Drishti (Version 2.4), Adobe Photoshop CC2018, Microsoft Office 2016, Blender 2.90

For manuscripts utilizing custom algorithms or software that are central to the research but not yet described in published literature, software must be made available to editors and reviewers. We strongly encourage code deposition in a community repository (e.g. GitHub). See the Nature Research guidelines for submitting code & software for further information.

Data

Policy information about availability of data

All manuscripts must include a data availability statement. This statement should provide the following information, where applicable:

- Accession codes, unique identifiers, or web links for publicly available datasets
- A list of figures that have associated raw data
- A description of any restrictions on data availability

The raw CT data generated in this study have been deposited in Zenodo (open access) [http://doi.org/10.5281/zenodo.4782778]

Field-specific reporting

Ecological, evolutionary & environmental sciences study design

All studies must disclose on these points even when the disclosure is negative.

Study description	We applied micro-CT and 3D rendering techniques to reveal novel information preserved inside the fossil specimens from the early Cambrian Chengjiang Biota. Other methods include light photography to document morphological information of the animals from the surface of the fossil slabs.
Research sample	Well pyritised arthropod fossils from the early Cambrian Chengjiang Biota, China
Sampling strategy	Well-pyritised samples were selected for this study due to the high possibility of getting good scanning results because of the density difference between the pyrite and matrix. We included data from multiple specimens of Leanchoilia illecebrosa to replicate results from the specimen in Figure 1. Because each specimen bears the same structures on many legs a few complete specimens in which the legs have been digitally dissected provide replication.
Data collection	Specimens were photographed and CT-scanned. Digital data were collected from the CT scanner and further processed on a powerful workstation with an open-domain software, Drishti 2.4. CT data were cleaned and digitally dissected with the software, and high-resolution images were then generated.
Timing and spatial scale	Fossil specimens used in this study were collected near Kunming, China, over a time span of several years (2015-2020). Selected specimens were scanned and the data were collected and analyzed during 2018-2020.
Data exclusions	All scanning data of the studied specimens here are included.
Reproducibility	No experiment is involved in the present study. 3D models have been reproduced on different computers with various software (e.g. Dragonfly), and comparable results have been produced.
Randomization	All samples involved in the study have been identified as existing species based on their diagnostic features.
Blinding	Blinding was not relevant to the present study, as all the studied samples were long known species.

Did the study involve field work? ☒ Yes ☐ No

Field work, collection and transport

Field conditions	During fossil collection in the field, temperature remained relatively stable, between 21-26 degrees. There was no rain during the collection of samples.
Location	Samples were collected from well-exposed outcrops near Kunming (Mafang and Ercaicun, Haikou county). See Material in main text for geographic coordinates.
Access & import/export	All the work involved in the study is in compliance with the local and national laws on fossil collections and storage. No import/export of samples was involved, as the samples remain in Yunnan Province.
Disturbance	Minimal. Manual collection was done rather than machine excavation, with selective sampling of stratigraphic horizons rather than bulk sampling.

Reporting for specific materials, systems and methods

We require information from authors about some types of materials, experimental systems and methods used in many studies. Here, indicate whether each material, system or method listed is relevant to your study. If you are not sure if a list item applies to your research, read the appropriate section before selecting a response.

Materials & experimental systems

n/a	Involved in the study	
☒	☐	Antibodies
☒	☐	Eukaryotic cell lines
☐	☒	Palaeontology and archaeology
☒	☐	Animals and other organisms
☒	☐	Human research participants
☒	☐	Clinical data
☒	☐	Dual use research of concern

Methods

n/a	Involved in the study	
☒	☐	ChIP-seq
☒	☐	Flow cytometry
☒	☐	MRI-based neuroimaging

Palaeontology and Archaeology

Specimen provenance	Since May 2004, the Yunnan Key Laboratory of Palaeobiology has been granted a long-term permission for Chengjiang fossil collection from local authorities such as the Science Department of Yunnan Province. This covers all of Yunnan Province.
Specimen deposition	Yunnan Key Laboratory for Palaeobiology, Yunnan University
Dating methods	The standard trilobite biostratigraphic scheme is used to recognise Chengjiang as Cambrian Series 2, Stage 3 (Eoredlichia-Wutingaspis biozone).

☒ Tick this box to confirm that the raw and calibrated dates are available in the paper or in Supplementary Information.

Ethics oversight	As no living samples are involved in the study, no ethical approval was required.

Note that full information on the approval of the study protocol must also be provided in the manuscript.
